# Phenotypic and genetic parameters of circadian rhythms from core body temperature profiles and their relationships with beef steers’ production efficiency profiles during successive winter feeding periods

**DOI:** 10.3389/fgene.2023.1026601

**Published:** 2023-01-19

**Authors:** Obioha Durunna, Jeffery A. Carroll, Jeff W. Dailey, Daalkhaijav Damiran, Kathy A. Larson, Edouard Timsit, Rex Parsons, Ghader Manafiazar, Herbert A. Lardner

**Affiliations:** ^1^ Department of Applied Research, Lakeland College, Vermilion, AB, Canada; ^2^ Department of Animal and Poultry Science, University of Saskatchewan, Saskatoon, SK, Canada; ^3^ USDA ARS Livestock Issues Research Unit, Lubbock, TX, United States; ^4^ Department of Agricultural and Resource Economics, University of Saskatchewan, Saskatoon, SK, Canada; ^5^ Department of Production Animal Health, Faculty of Veterinary Medicine, University of Calgary, Calgary, AB, Canada; ^6^ Australian Centre for Health Services Innovation and Centre for Healthcare Transformation, School of Public Health and Social Work, Faculty of Health, Queensland University of Technology, Kelvin Grove, QLD, Australia; ^7^ Animal Science and Aquaculture Department, Faculty of Agriculture, Dalhousie University, Halifax, NS, Canada

**Keywords:** winter, core body temperature, reticulo-rumen, rectal, feed efficiency, telemetry, genetic parameters, multi-environment evaluations

## Abstract

This 2-year study evaluated differences in circadian parameters obtained from measures of core body temperatures using telemetric reticulo-rumen and rectal devices during two winter feeding regimes in western Canada. The study also estimated phenotypic correlations and genetic parameters associated with circadian parameters and other production traits in each feeding regime. Each year, 80 weaned steer calves (initial age: 209 ± 11 days; BW: 264 ± 20 kg) from the same cohort were tested over two successive regimes, Fall-Winter (FW) and Winter-Spring (WS) at Lanigan, Saskatchewan, Canada. The steers received forage-based rations in both regimes where the individual feed intake was measured with automatic feeding units. During the trial, the reticulo-rumen (RTMP) and rectal (RCT) temperatures were simultaneously measured every 5 min using telemetric devices. These were used to calculate the circadian parameters (Midline Estimating Statistic Of Rhythms, amplitude, and acrophase/peak time) for both temperature measures. Growth and efficiency performance traits were also determined for all steers. Each steer was assigned into inefficient, neutral, and efficient classes based on the SD of the residual feed intake (RFI), residual gain (RG), and residual intake and gain (RIG) within each year and feeding regime. Higher (*p* < 0.0003) RTMP and rectal temperature MESORs were observed in the Fall-Winter compared to the Winter-Spring regime. While the two test regimes were different (*p* < 0.05) for the majority of the RTMP or RCT temperature parameters, they did not differ (*p* > 0.10) with the production efficiency profiles. The heritability estimates were higher in FW (0.78 ± 0.18 vs. 0.56 ± 0.26) than WS (0.50 ± 0.18 vs. 0.47 ± 0.22) for the rumen and rectal MESORs, respectively. There were positive genetic correlations between the two regimes for the RTMP (0.69 ± 0.21) and RCT (0.32 ± 0.59). There was a negative correlation (*p* < 0.001) between body temperature and ambient temperature. The high heritability estimates and genetic correlations for rumen and rectal temperature parameters demonstrate their potential as beef genetic improvement tools of economic traits associated with the parameters. However, there are limited practical implications of using only the core-body temperature as a proxy for production efficiency traits for beef steers during winter.

## Introduction

The recent advancements in telemetry or wireless data transfer systems have made it easier and cheaper to automate and acquire difficult-to-measure data, such as rumen temperature, from individual cattle. Compared to conventional manual body temperature measurements, these telemetric systems bolster the collation in the collection of individual temperature information from large cohorts under different environmental conditions, especially in the Canadian prairies where the extreme cold weather may limit or confound manual data collection activities. Here, we evaluate the potential of capturing, analyzing, and using sensor-generated data to improve the assessments and selection of replacement candidates during western Canadian winters.

The frigid winter conditions in the northern plains increase beef production costs due to limited forage growth and the need for extra feed to meet elevated energy requirements for body maintenance. Cold stress could reduce beef cattle’s daily body gains and feed efficiency by 10% and 5%, respectively (Hoelscher, 2001). Even though the cattle breeds raised in these regions have acclimatized to subzero temperatures, the winter seasons considerably impact individual feed/growth efficiency performance. This period coincides with the backgrounding period of calves raised under the early-calving production systems (Durunna et al., 2014). Performance testing for feed or growth efficiency also occurs within this period for those earlier-weaned calves.

Accurate assessments of these production efficiency profiles using residual feed intake (RFI) or residual body gain (RBG) require individual animal intake and growth information (BIF Guidelines Wiki, 2021), which are labor-intensive or costly to collect. The feed tests are cost-prohibitive, costing over $400 USD per head for a standard 91-day performance test (including the acclimatization period). Individual variations due to seasons may demand multi-tests and a more robust selection of replacements across different environmental conditions (Durunna et al., 2011). At this cost, multiple tests on potential candidates are not practical for most beef producers. The availability of cheaper indicator traits will encourage multi-period testing and enable beef producers to identify better replacement candidates. Regularly assessing these production efficiency traits in replacement candidates will improve the beef industry’s profitability and sustainability.

It has been shown that many factors influence feed efficiency (assessed by RFI), such as physical activity, tissue metabolism, protein turnover, and heat increment of feeding (Richardson and Herd, 2004), all of which are thermogenic mechanisms. Previous studies (Richardson and Herd 2004; Nkrumah et al., 2006) have reported that heat production accounts for a large proportion of variation in RFI. Other studies have demonstrated that radiated heat (measured *via* infrared thermography) from the skin can predict the feed efficiency profiles of cattle (Schaefer et al., 2005; Montanholi et al., 2009). However, the results were affected by peripheral factors such as body location of the infrared measurement, animal handling, and environmental conditions (such as wind speed and solar loading), which resulted in low repeatability across test days. On the other hand, other studies have shown that reticulo-rumen temperature (RTMP) is more reliable under unstable environmental conditions when compared to subdermal locations (Hahn et al., 1990; Carroll et al., 2009; Reuter et al., 2010).

Further, deploying new phenotypes (from advanced analytical methods) toward predicting outcomes associated with difficult-to-measure, complex, or relevant economic traits will advance the beef industry. Specifically, this study evaluated if cattle with different production efficiency profiles have different circadian temperature rhythms (CTR) during these regimes. The study also assessed the correlations between production efficiency measures and core body temperature (CBT; measured remotely from the reticulo-rumen and rectum) during two successive winter test regimes to determine whether an animal’s CBT can predict the production efficiency profile in cold environments. The estimated phenotypic and genetic parameters associated with each CTR feature will help decide whether or not they are practical screening or selection tools for the North American beef industry.

## Materials and methods

### Test animals, experimental site, and test regimes

All animals in this study were managed according to the guidelines of the Canadian Council of Animal Care (CCAC, 2009). The experimental procedures were approved by the University of Saskatchewan Animal Care Committee (Protocol No. 20090107).

Each year, eighty spring-born (birthdate: April to late May) Angus steers were recruited for two successive feeding trials at the Western Beef Development Centre’s (WBDC) Termuende Research Ranch near Lanigan (lat. 51°51′N, long. 105°02′W), Saskatchewan, Canada. The first feeding regime in 2016–2017 of Fall-Winter (FW) ran from 16 November 2016 to 9 February 2017 (total = 85 days), while the second feeding regime of Winter-Spring (WS) ran from 23 February 2017 to 12 May 2017 (total = 78 days). The FW for the second year (2017–2018) of the study was from 21 November 2017, to 14 February 2018 (total = 85 days), while the WS ran from 28 February 2018 to 17 May 2018 (total = 78 days). For data analyses, the average valid days were 78 (4) d and 65 (4) d for the FW and WS regimes, respectively.

The daily weather information was obtained from the Environment Canada weather data repository (www.climate.weatheroffice.ec.gc.ca) for Watrous, Saskatchewan, approximately 50 km southeast of the experiment site. Daily temperature-humidity indices were calculated from the daily dry bulb temperature and relative humidity information (Armstrong 1994).

The initial average age and body weight (SD) for Year1-FW, Year1-WS, Year2-FW, and Year2-WS, were 209 (10), 308 (10), 210 (11), and 309 (11) d, respectively; while the average initial body weights (SD) were 262 (16), 348 (21), 266 (20) and 344 (28) kg, respectively. All steers were vaccinated against the Bovine Respiratory Syncytial Virus, Bovine Viral Diarrhea, Infectious Bovine Rhinotracheitis, and Parainfluenza 3 (Express 5; Boehringer Ingelheim Vetmedica, Inc. St. Joseph, MO). The animals also received a Clostridium 8-way modified live vaccine (Covexin 8; Schering-Plough Animal Health, Guelph, Ontario, Canada) and a 36 mg Zeranol implant (RALGRO®; Schering-Plough corp., Kenilworth, NJ, Unioted States).

The steers were stratified by body weight and randomly assigned to two pens with size of 50 × 120 m each. Each pen was fitted with eight (8) GrowSafe® feed bunks (GrowSafe Systems Ltd., Airdrie, Alberta, Canada) that measured each steer’s individual daily feed intake. The Growsafe® system consists of a radio frequency identification tag, feeding troughs, a data reader panel, and a computer. Each animal wore a transponder embedded in an ear tag (Allflex USA Inc., Dallas/Fort Worth, TX), such that the radio waves emitted by the transponder are detected as each animal feeds from each trough. Each of the eight feeding bunks in each pen rested on two load bars, such that feed consumed from any bunk was assigned to the animal present at that bunk. The feeding information is logged in the reader panel before being wirelessly transmitted to a computer equipped with the data acquisition software.

All steers received a 21-day acclimatization period, allowing them to adapt to the feeding environment. During this time, the steers learned to access feed and water from the automatic feeding systems and heated water troughs. The body weights were collected on two consecutive days at the beginning, 2-week intervals during the test, and end of each feeding regime in each year. Woodchips were provided as bedding materials while the ultrasound backfat thickness was collected (Bergen et al., 1997) at the beginning and end of each feeding regime using an Aloka 500 V real-time ultrasound machine (3.5 MHz; Aloka Inc., Wallingford, CT), equipped with a 17-cm linear array transducer.

### Feeding management and diet quality

The steers received forage-based diets in both regimes within each year of the study, where the FW had a higher forage content than the WS. A ration balancing program (CowBytes Version 5, Alberta Agriculture, Food and Rural Development, Alberta, Canada) was used to formulate the diets based on bodyweight, forage nutrient analysis, and environmental conditions. The FW diet comprised 70.2% processed bromegrass-alfalfa hay, 29.4% rolled barley, and 0.4% pelleted supplement, while the WS diet consisted of 56.3% bromegrass/alfalfa hay, 37.9% barley grain, 1.3% barley straw, and 4.5% pelleted supplement. The steers were adapted to the WS diet in three steps by increasing the portion of barley grain in the total mixed ration while reducing the hay content. Mixed-feed was delivered twice daily at 0800 and 1,500 h throughout the feeding regimes using a Farm Aid Mixer Wagon equipped with a digital scale (model 430, Corsica, SD). The steers also had access to a commercial 2:1 mineral (Cargill ‘Right Now Emerald’) that contained 22% Ca, 14% P, 1% Zn, 125 mg/kg I, 4,000 mg/kg Cu, 5,300 mg/kg Mg, 40 mg/kg Co, 450 mg/kg Fe, 200 KIU/kg of vitamin A, 40 IU/kg of vitamin E and cobalt iodized salt block (99% NaCl).

The ingredient and mixed diets were sampled every 14 days to be evaluated for DM (method 930.15; AOAC International, 1990) by drying them in paper bags within a forced air oven at 55°C for 72 h. The dried samples were then ground to pass through a 1-mm screen using a Wiley mill (Model 4, Arthur H. Tomas Co., Philadelphia, PA) for further analyses, which included ash (method 942.05; AOAC International, 1990), crude fat (method 920.02; AOAC International, 1990), and CP (method 984.13; AOAC International, 1990). The acid detergent fiber (ADF) and neutral detergent fiber (NDF) fractions were determined with heat-stable α-amylase (Van Soest et al., 1991) using an ANKOM Fiber Analyzer (ANKOM Technology Corporation., Fairport, NY). Other analyses included starch (Hall 2009), ash (method 942.05; AOAC International, 2000), fat (method 2003.05; AOAC International, 2000) using a Tecator® extraction unit, and minerals (method 985.01; AOAC International, 2000).

The net energy for maintenance (NEm) and net energy for gain (NEg) were calculated according to NRC Beef (2000). Non-fiber carbohydrate (NFC) was calculated based on Linn (2003) as: NFC, % = 100–(CP, % + Fat, % + Ash, % + NDF, % + NDICP, %) where NDICP is neutral detergent insoluble crude protein. The TDN and DE levels were determined using methods described by Weiss et al. (1992), while Nem and Neg were estimated using the NRC Beef Model (1996).

### Production traits and efficiency calculations

The traits measured or calculated in this study include dry matter intake (DMI), average daily gain (ADG), feed conversion ratio (FCR), feed conversion efficiency (FCE), RFI, RBG, and residual intake and gain (RIG). The individual DMI was calculated from the feed intake measured with the GrowSafe® (GrowSafe Systems Ltd., Airdrie, Alberta, Canada) described by Durunna et al. (2011). These DMI within each regime were converted to metabolizable energy intake equivalents (MEI) and standardized to 10 MJ ME kg^−1^ DM.

The ADG was determined by regressing individual body weights (collected every 2 weeks) on days, and the regression coefficient is considered ADG. The FCR was calculated as the ratio of DMI to ADG, while the FCE was the ratio of ADG to DMI. The mid-test BW was converted to metabolic body weight (MWT) by BW^0.75^. The RFI and RBG were the residuals from Eqs 1, 2, as shown below:

$${DMI}_{j}=\beta_{0}+\beta_{1}{ADG}_{j}+\beta_{2}{MWT}_{j}+\beta_{3}{BKFT}_{j}+\varepsilon_{j},$$ (1)

$${ADG}_{j}=\beta_{4}+\beta_{5}{DMI}_{j}+\beta_{6}{MWT}_{j}+\beta_{7}{BKFT}_{j}+\varepsilon_{j},$$ (2)

where DMI_j_ is the average standardized MEI for the *j*th steer during the test regime, β0 is the regression intercept for model 1, β1 is the ADG regression coefficient for the *j*th steer, β2 is the MWT regression coefficient for the *j*th steer, β3 is the regression coefficient for the difference in backfat thickness for the *j*th steer, and ε_j_ (in model 1) indicates the residuals (as RFI). Similarly, for model 2, ADG_j_ is the actual ADG for each animal calculated by linear regression model, β_4_ is the regression intercept for model 2, β5 is the DMI regression coefficient, β7 is the MWT regression coefficient, β6 is the regression coefficient for the difference in backfat thickness, and ε_j_ indicates the RBG. The RFI and RBG were standardized with the respective standard deviations within each regime and then incorporated into model 3 to calculate RIG.

$${RIG}_{j}=-1\times {RFIs}_{j}+{RBGs}_{j}$$ (3)

Where RFIs and RBGs are standardized values from Eqs 1, 2, respectively. The steers were then classified into efficient, neutral, and inefficient classes for RFI, RBG, and RIG based on 0.5 SD from the mean in order to determine common characteristics from members in each class.

### Reticulo-rumen and rectal temperatures

The steers’ reticulo-rumen temperatures (RTMP) were continuously measured using telemetric boluses (Thermobolus®, Capteur San’Phone, Medria, Châteaubourg, France). The Thermobolus® system was described by Timsit et al. (2011) in detail. In short, the main components for such telemetric temperature systems include the sensor equipped with a transducer that can detect heat in a known physical form and translate such signal to a decoder or data collection system that interprets the signal into readable information (Godyń et al., 2019). Before administering each bolus orally to calves, the accuracies of the boluses were evaluated *in vitro via* a circulating water bath (Anova Precision® Cooker, San Francisco, CA, United States) over a temperature range of 39.3–39.5°C. Each steer received a bolus before the 21-day adaptation phase. Each bolus was programmed to measure the RTMP every 5 min throughout the test regime and wirelessly transmit the data to a base station connected to the internet. The base station was located inside a temperature-controlled barn, approximately 15 m away from the steers’ pens. The data transmitted from all steers were stored on a server to pre-process the raw data using autoregressive modeling (order 4) and adaptive filtering. The pre-processing was conducted to eliminate the effect of drinking bouts (Timsit et al., 2011).

The rectal temperature (RCT) devices (Reuter et al., 2010) were installed on 40 randomly selected steers. The rectal devices were fastened to selected steers after the 21-day warm-up period and dismantled after approximately 4 weeks in each regime. Each RCT device comprised an aluminum tail harness, a temperature logger (length = 25.4 mm, diameter = 8.3 mm, 3.3 g; DST Micro-T®, Star-Oddi, MeterMall United States, Marysville, OH, Umited States), and polyethylene tubing (length = 21 cm; outer diameter = 0.95 cm). The loggers were also programmed to collect the rectal temperatures every 5 min, synchronized with the rumen devices. The RTMP and RCT data were filtered to remove values below 32°C or above 42°C.

The cattle body temperature oscillates every 24 h (Halberg, 1959) and can be characterized by daily circadian parameters, including the MESOR (Midline Estimating Statistic Of Rhythms), amplitude, and acrophase. It was important to calculate the daily average temperature while accounting for the oscillations. These parameters were estimated for each steer with the circa_single() function from the circacompare package (Parsons et al., 2020; https://rdrr.io/cran/circacompare/src/R/circa_single.R) in R (R Core Team 2021). The function fits a non-linear least-squares model (model 4 below) to the data from a subject or group.

$$Y\;∼\;k+\alpha \times \cos\left(т\_r\;–\;\Phi \right)$$ (4)

where y is the outcome or response variable, k is the MESOR or midpoint of the cosine rhythm, α is the amplitude of the rhythm, т_r is the time (in radians which was converted from hours assuming a 24-h) that the temperature cycle is at its peak, while Φ is the phase which represents the difference between the time of peak levels from the reference time (midnight) in radians.

The MESOR represents each individual’s midpoint of daily (rumen or rectal) temperature rhythm. The amplitude is half of the difference between the peak and trough of either the rumen or rectal temperature cycle, reflecting the difference between the MESOR and either extreme of the oscillation range within the 24-h period. The time at which the variable of interest peaks is known as the acrophase. Days with no rhythmicity (*p* > 0.05) were excluded from the analysis. The least-squares means for daily rumen and rectal temperature parameters were derived for each steer within each regime. Like the feed-efficiency classification, the study evaluated whether differences in production traits existed when each steer’s MESOR was classified as high, medium, or low based on 0.5 SD (>0.5 SD; ±0.5 SD; <0.5 SD, respectively) within each regime.

### Statistical analyses and genetic parameters associated with temperature/circadian parameters

Differences between the two regimes for body weight, DMI, ADG, production efficiency measures and temperature parameters were analyzed using SAS® software Proc Mixed (SAS Inst. Inc., Cary, NC) with a model that included the year, feeding regime, and year-regime interaction. The initial weight at the trial’s start was used as a covariate. To determine the differences in the rumen and rectal temperature parameters among the production efficiency classes (RFI, RBG, and RIG), similar models which also included the appropriate efficiency class (as a fixed effect) and individual animals (within each regime) as a random effect was employed. The relationships between the rumen and rectal temperatures and other production performance indicators were evaluated using SAS® software’s Correlation Procedure. Partial correlations (which adjusted for daily information from individual animals) determined the relationships between ambient temperature and either rumen or rectal MESOR.

Pedigree information was available for all steers. Dams were matched to calves at calving while the sires were matched to potential calves through DNA paternity tests to identify potential sires from multi-sire breeding groups. About 33% of the sires (8 of 24) were common between both years while about 14% (19 of 138) dams were common between both years. The variance-covariance components, the heritability of the traits of interest, and their genetic and phenotypic correlations were determined using bivariate models that assumed each regime as a different trait for the temperature and circadian parameters. All parameters were estimated using Bayesian procedures THRGIBBS1F90 (Misztal et al., 2015) to generate aposteriori distributions of 3,600 samples for every 250 cycles from 1,000,000 iterations recognizing a burn-in period of 100,000 iterations. The pen, feeding regime, and year effects were included as fixed factors, while the initial weight was assigned a covariate. The analyses for rectal temperature parameters included only the steers that received the rectal device.

## Results

Table 1 shows the nutrient composition of the steers’ diets in the two regimes. The WS had higher dry matter, but both regimes had similar CP and TDN contents. The rumen bolus varied within ±0.05°C (CV = 0.12%), showing the ability of the tool to relay correct temperature measures when immersed in liquid environments. No rumen bolus was regurgitated or excreted throughout the study. However, four rectal dataloggers were lost in the pens because they were dislodged from the rectal mount. Some steers showed low-temperature readings due to partially- or wholly-protruded polyethylene tubings (which supported the dataloggers). Such low readings were excluded, along with the data from steers having less than 80% of the complete rectal data.

**TABLE 1:** Nutrient composition of diet fed to backgrounding steers during the Fall-Winter and Winter-Spring regimes over the 2 years of study.

Item	Regime 1 high-forage diet	Regime 2 moderate-forage diet
Dry matter (%)	80.67 ± 14.00	85.48 ± 12.00
Ash (%)	6.67 ± 0.29	8.60 ± 1.10
Crude fat (%)	1.27 ± 0.47	1.62 ± 0.29
Acid detergent fibre (%)	40.60 ± 0.86	37.89 ± 2.72
Neutral detergent fibre (%)	60.83 ± 2.08	56.54 ± 3.33
Non-structural carbohydrate profile (%)	16.32 ± 3.54	21.17 ± 3.69
Starch (%)	3.75 ± 0.94	7.08 ± 3.21
Crude protein (%)	11.35 ± 1.79	10.80 ± 1.83
Total digestible nutrients^a^, % DM	57.27 ± 0.67	59.38 ± 2.12
ME (mJ)^b^ = TDNx0.04409 × 0.82 × 4.184	8.66	8.98
Energy values (Mcal/kg DM)		
Net energy for gain	0.65 ± 0.02	0.72 ± 0.06
Net energy maintenance	1.37 ± 0.02	1.44 ± 0.06
Macro elements (%, DM)		
Calcium	0.64 ± 0.13	1.25 ± 0.35
Phosphorus	0.17 ± 0.04	0.19 ± 0.04
Potassium	1.56 ± 0.15	1.53 ± 0.32
Sulphur	0.15 ± 0.02	0.16 ± 0.01
Magnesium	0.21 ± 0.03	0.23 ± 0.04
Sodium	0.02 ± 0.01	0.14 ± 0.07
Micro minerals (g/kg DM)		
Zinc	15.27 ± 2.93	63.33 ± 27.33
Iron	85.32 ± 21.97	173.81 ± 106.09
Manganese	40.20 ± 5.84	68.20 ± 15.37
Copper	4.68 ± 0.35	15.71 ± 7.45

^a^ Calculated using the Weiss equation (1992).

^b^ ME, MJ∙kg^−1^ of DM = [(TDN, %/100) × 4.4 Mcal∙kg^−1^ of TDN] × 4.184 MJ of DE∙Mcal^−1^ × 0.82 MJ of ME∙MJ^−1^ of DE (National Research Council, 1996).

**FIGURE 1:**
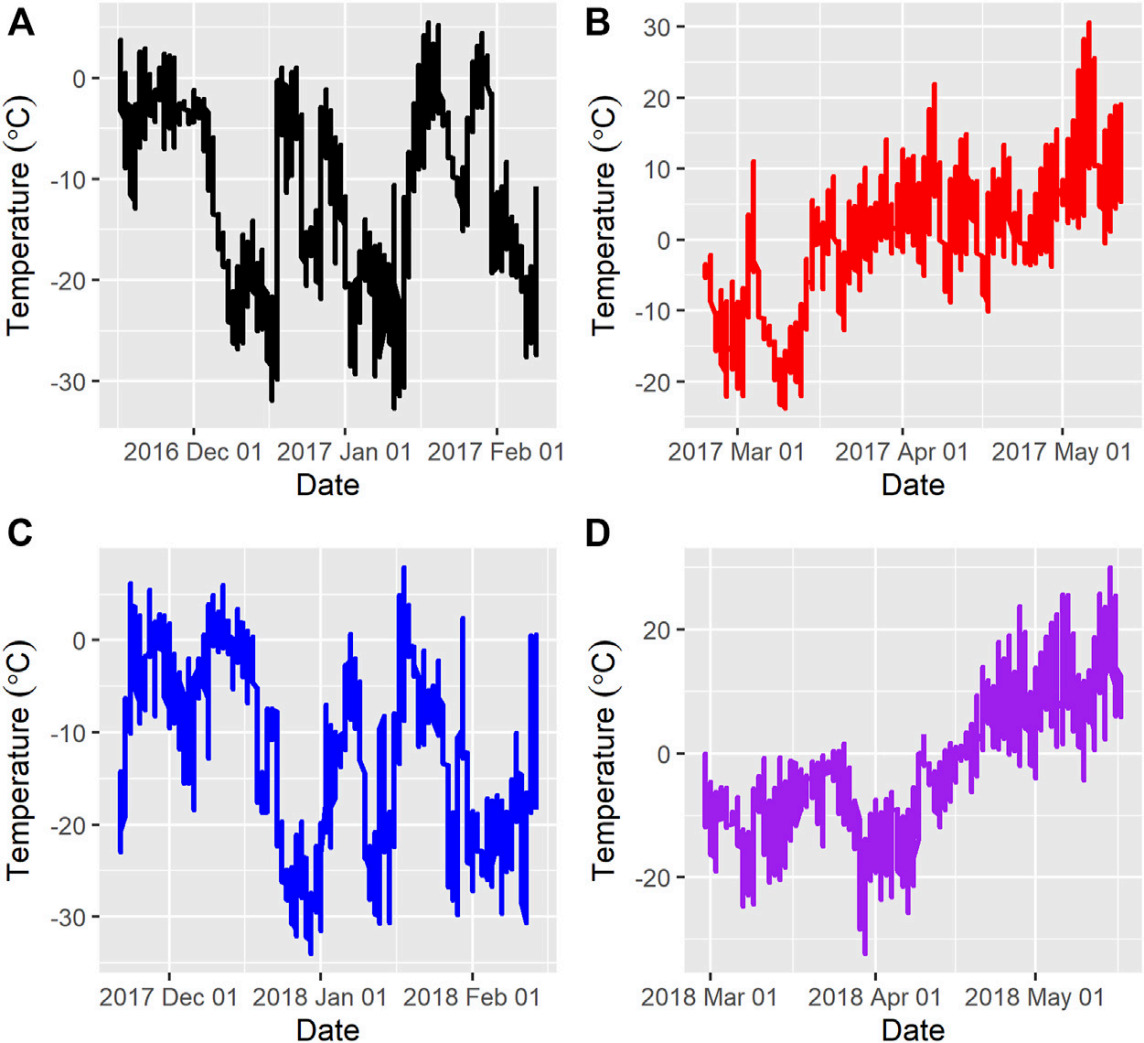
Average ambient temperatures during the feeding trials **(A)** = Year 1, feeding regime 1 (Fall-Winter); **(B)** = Year 1, feeding regime 2 (Winter-Spring); **(C)** = Year 2, feeding regime 1 (Fall-Winter); **(D)** = Year 2, feeding regime 2 (Winter-Spring).

**FIGURE 2:**
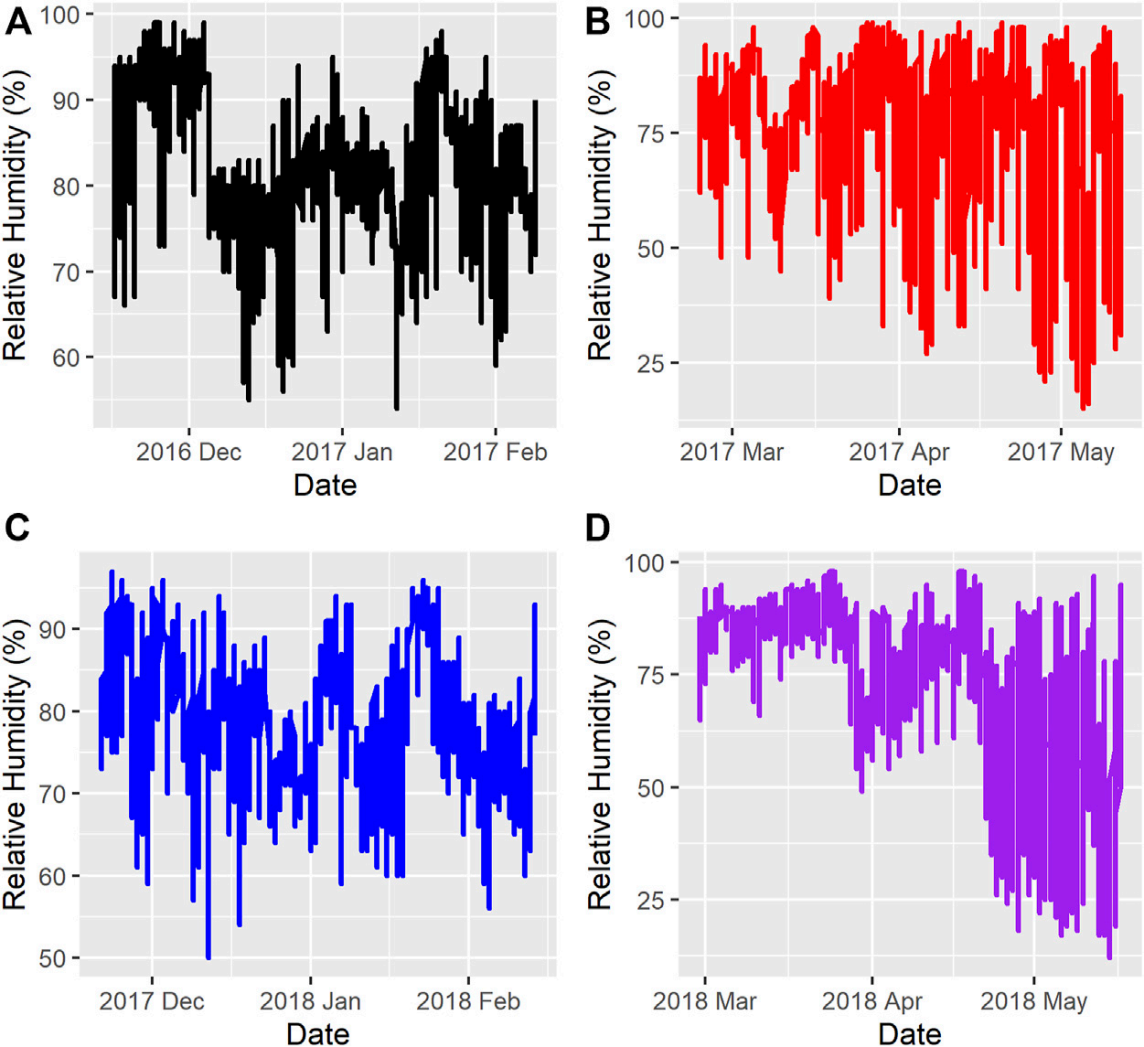
Relative Humidity during the feeding trials **(A)** = Year 1, feeding regime 1 (Fall-Winter); **(B)** = Year 1, feeding regime 2 (Winter-Spring); **(C)** = Year 2, feeding regime 1 (Fall-Winter); **(D)** = Year 2, feeding regime 2 (Winter-Spring).

Figure 1 and Figure 2 show the ambient temperatures and humidity within all years and regimes. There was no difference between the 2 years for the average ambient temperature (*p* > 0.89) or relative humidity (*p* > 0.85) for either the FW or WS. The average ambient temperature and humidity in the first year were -11.31 ± 0.22°C; 82.15 ± 0.33% and 0.28 ± 0.23°C; 73.50 ± 0.35% for the FW and WS, respectively. In the second year, the FW and WS had −12.15 ± 0.22°C; 78.94 ± 0.33% and −1.91 ± 0.23°C; 72.54 ± 0.35%, respectively.

The production performance outcomes and circadian temperature parameters are shown in Table 2. There was no regime-by-year interaction (*p* > 0.05) for the average age and weight at the initiation of the study, DMI and for all temperature parameters except the acrophase of rumen MESOR (*p* < 0.0001). As expected, the DMI and MEI were greater (*p* < 0.0001) in the WS compared to the FW. While there were no differences between the two regimes (*p* > 0.10) for RFI and RIG, however, RG showed differences (*p* = 0.003) between the regimes. The FCR was greater (*p* < 0.0001) in the FW, while the FCE was greater (*p* < 0.0001) in the WS.

**TABLE 2:** Production performance and circadian parameters observed within each feeding regime over the 2 years of the study.

Variable	Fall-winter	Winter-spring	*p*-values			
			Regime	Year	Regime x year	Initial weight
SOT Age (d)	211.34 ± 1.41	305.9 ± 1.42	<0.0001	0.32	0.89	0.053
SOTWT (kg)	260.4 ± 1.75	342.1 ± 1.76	<0.0001	0.006	0.86	
EOT WT (kg)	328.3 ± 2.13	442.7 ± 2.14	<0.0001	0.15	0.009	
DMI (kg d^−1^)	9.12 ± 0.11	10.09 ± 0.11	<0.0001	0.08	0.18	<0.0001
MEI (MJ kg^−1^ DM)	7.90 ± 0.10	9.05 ± 0.10	<0.0001	0.18	0.55	<0.0001
ADG (kg d^−1^)	0.85 ± 0.02	1.23 ± 0.02	<0.0001	0.98	<0.0001	0.004
FCR	11.03 ± 0.20	8.40 ± 0.20	<0.0001	0.14	<0.0001	0.81
FCE	0.09 ± 0.002	0.12 ± 0.002	<0.0001	0.73	<0.0001	0.52
RFI (kg DM d^−1^)	-0.02 ± 0.08	0.02 ± 0.08	0.80	0.99	0.98	0.77
RG (kg d^−1^)	-0.04 ± 0.02	0.04 ± 0.02	0.003	0.96	0.76	0.0008
RIG	-0.32 ± 0.21	0.33 ± 0.22	0.10	0.98	0.86	0.06
Rumen MESOR	39.80 ± 0.02	39.68 ± 0.02	0.0002	<0.0001	0.25	0.29
Amplitude	0.24 ± 0.010	0.29 ± 0.012	0.0002	0.004	0.23	<0.0001
Acrophase/Peak time	16.21 ± 0.23	18.81 ± 0.27	<0.0001	0.01	<0.0001	0.18
Rectal MESOR	39.28 ± 0.02	38.96 ± 0.03	<0.0001	0.02	0.25	0.07
Amplitude	0.25 ± 0.01	0.30 ± 0.02	0.04	0.37	0.08	0.53
Acrophase/Peak time	17.45 ± 0.28	19.19 ± 0.31	0.0004	<0.0001	0.27	0.67

SOT, start of test; BWT, body weight; EOT, end of test; DMI, dry matter intake; Regime, Feeding Regime; MEI, metabolizable energy intake; ADG, average daily gain; FCR, feed conversion ratio; FCE, feed conversion efficiency; RFI, residual feed intake; RBG, residual body gain; RIG, residual intake and gain.

There were differences (*p* < 0.05) between the two regimes for all the rumen and rectal circadian temperature parameters. The MESOR for both rumen and rectal temperatures was greater in the FW. The rumen MESORs were 39.75°C, 39.65°C, 39.82°C, and 39.75°C, respectively, for Yr1 FW, Yr1 MS, Yr2 FW, and Yr2 MS. Similarly, the rectal MESOR for the same regimes were 39.28, 38.89, 39.37, and 38.94°C, respectively. The FW had lower values for amplitude, while the acrophase for either the ruminal (18.8 vs. 16.2 h) or rectal (19.2 vs. 17.5 h) temperature occurred later during the WS compared to the FW for all efficiency classifications. The correlation between average rumen temperature and the average ambient temperature was -0.29 (*p* = 0.0009), while the correlation between the average rectal temperature and average ambient temperature was −0.77 (*p* < 0.0001).

Production efficiency classifications (using standard deviation units) provide a platform to differentiate animal groups having similar characteristics. The rumen amplitude differed (Table 3, *p* = 0.03) for all the efficiency classification groups, but there were no differences (*p* > 0.05) among the efficient, neutral and inefficient classes for the majority of the circadian parameters for either the rumen or rectal temperature when compared under the RFI, RG or RIG classifications.

**TABLE 3:** Production efficiency-based classification (LSmeans) and circadian parameters for rumen and rectal temperature parameters calculated within the two regimes.

Variable	Efficient	Neutral	Inefficient	Class	Regime	Year	Class*Regime
RFI Classification							
Rumen Temperature							
Mean Temperature (MESOR)	39.72 ± 0.01	39.74 ± 0.01	39.75 ± 0.01	0.30	0.0004	<0.0001	0.72
Amplitude	0.26 ± 0.009	0.26 ± 0.008	0.27 ± 0.009	0.31	0.0003	0.005	0.03
Acrophase/Peak time	17.72 ± 0.19	17.32 ± 0.18	17.56 ± 0.19	0.03	<0.0001	0.01	0.31
Rectal Temperature							
Mean Temperature (MESOR)	39.11 ± 0.03	39.10 ± 0.03	39.15 ± 0.03	0.34	<0.0001	0.02	0.57
Amplitude	0.27 ± 0.01	0.29 ± 0.01	0.27 ± 0.01	0.56	0.08	0.31	0.09
Acrophase/Peak time	18.43 ± 0.25	18.15 ± 0.24	18.42 ± 0.24	0.51	0.0006	<0.0001	0.99
RBG Classification							
Rumen Temperature							
Mean Temperature (MESOR)	39.75 ± 0.01	39.74 ± 0.01	39.73 ± 0.01	0.64	0.0004	<0.0001	0.94
Amplitude	0.26 ± 0.009	0.27 ± 0.009	0.27 ± 0.009	0.03	<0.0001	0.004	0.44
Acrophase/Peak time	17.50 ± 0.18	17.52 ± 0.19	17.50 ± 0.19	0.98	<0.0001	0.01	0.94
Rectal Temperature							
Mean Temperature (MESOR)	39.12 ± 0.03	39.14 ± 0.03	39.11 ± 0.02	0.75	<0.0001	0.03	0.42
Amplitude	0.28 ± 0.01	0.26 ± 0.01	0.29 ± 0.01	0.26	0.07	0.51	0.18
Acrophase/Peak time	18.42 ± 0.24	18.22 ± 0.26	18.30 ± 0.34	0.78	0.0004	<0.0001	0.30
RIG Classification							
Rumen Temperature							
Mean Temperature (MESOR)	39.73 ± 0.014	39.74 ± 0.012	39.74 ± 0.014	0.88	0.0004	<0.0001	0.68
Amplitude	0.26 ± 0.009^ab^	0.26 ± 0.009^a^	0.27 ± 0.009^b^	0.03	<0.0001	0.003	0.17
Acrophase/Peak time	17.62 ± 0.19	17.40 ± 0.18	17.53 ± 0.20	0.32	<0.0001	0.01	0.22
Rectal Temperature							
Mean Temperature (MESOR)	39.10 ± 0.03	39.13 ± 0.03	39.13 ± 0.03	0.66	<0.0001	0.02	0.76
Amplitude	0.28 ± 0.01	0.26 ± 0.01	0.28 ± 0.01	0.23	0.03	0.41	0.12
Acrophase/Peak time	18.40 ± 0.24	18.42 ± 0.24	18.19 ± 0.23	0.61	0.0004	<0.0001	0.17

RFI, residual feed intake; RBG, residual body gain; RIG, residual intake and gain. FW, Fall-Winter; WS, Winter-Spring regime.

### Relationships among the traits and parameters within and between the two regimes

**FIGURE 3:**
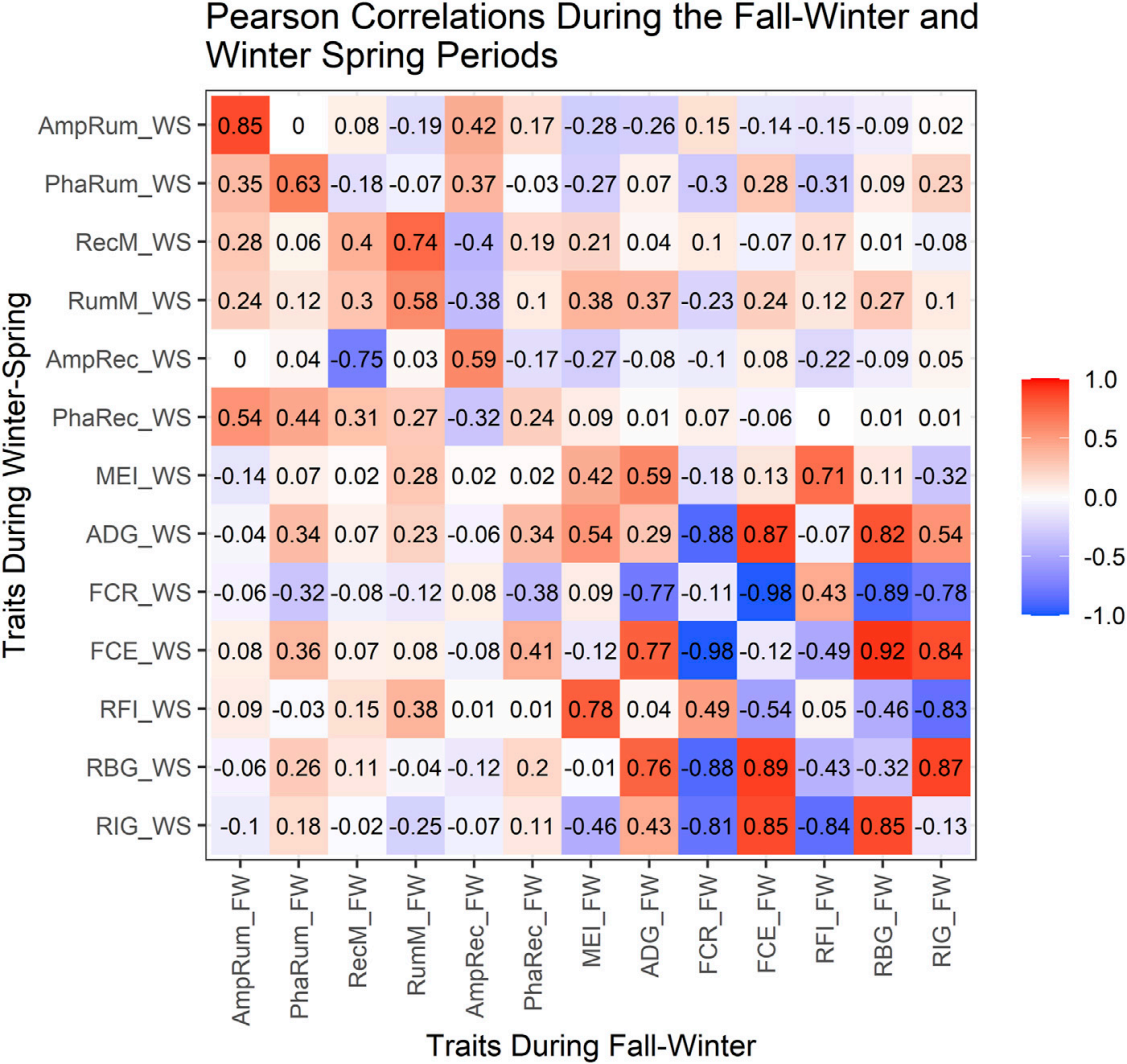
Fall-Winter and Winter-Spring Pearson correlations. FW, Fall-Winter regime; WS, Winter-Spring regime; AmpRum, Rumen amplitude; PhaRum, Rumen acrophase; RecM, Rectal MESOR; RumM, Rumen MESOR; AmpRec, Rectal amplitude; PhaRec, Rectal acrophase; MEI, Metabolizable energy intake; ADG, Average Daily Gain; FCR, Feed Conversion Ratio; FCE, Feed Conversion Efficiency; RFI, Residual Feed Intake; RBG, Residual Body Gain; RIG, Residual Intake and Gain; The diagonal (upper left to lower right) indicates the correlations between FW and WS regimes for the traits evaluated in the study, above the diagonal shows the correlations within the FW regime while below the diagonal shows the correlations within the WS regime

Figure 3 shows the correlations among production traits and rhythm-adjusted temperature parameters within each test-regime, and between the two regimes. Within the FW (above the diagonal), a strong positive correlation (*p* < 0.0001) was observed between the MESORs for rumen and rectal temperature (0.74) but was moderate between rumen amplitude and rectal amplitude (0.42; *p* < 0.001). The MEI was moderately positively correlated with rumen MESOR (0.38; *p* < 0.0001) but weaker with rectal MESOR (0.21; *p* < 0.10) during the FW. There was no relationship (r = −0.03; *p* = 0.84) between the acrophase for rumen and rectal temperatures during the FW, but the correlation was stronger (0.44; *p* = 0.0003) in WS (below the diagonal). The MEI showed weak correlations with the rumen (−0.14) or rectal (0.02) amplitudes.

**TABLE 4:** Heritability (±PSD) and some genetic parameters for circadian core body temperature parameters.

Variable	FW h^2^	HPD95	WS h^2^	HPD95	FW σg	WS σg	r_g_	r_p_
Rumen MESOR	0.78 ± 0.18	0.44–0.99	0.50 ± 0.18	0.17–0.85	0.013	0.007	0.69 ± 0.21	0.63 ± 0.05
Rectal MESOR	0.56 ± 0.26	0.09–0.99	0.47 ± 0.22	0.07–0.87	0.01	0.02	0.32 ± 0.59	0.03 ± 0.17
Bolus Amplitude	0.56 ± 0.25	0.13–0.99	0.39 ± 0.19	0.05–0.76	0.0008	0.0007	0.23 ± 0.52	0.41 ± 0.07
Rectal Amplitude	0.52 ± 0.27	0.06–0.98	0.42 ± 0.21	0.04–0.80	0.001	0.01	0.40 ± 0.59	-0.01 ± 0.16
Bolus Acrophase	0.17 ± 0.17	0.00002–0.54	0.34 ± 0.18	0.02–0.69	0.31	0.28	0.53 ± 0.59	0.30 ± 0.08
Rectal Acrophase	0.32 ± 0.25	0.0001–0.83	0.50 ± 0.22	0.12–0.91	0.62	1.26	0.14 ± 0.66	0.40 ± 0.13

FW, Fall-Winter regime; WS, winter spring; h^2^, heritability; HPD95, Highest posterior density intervals; r_g_, Genetic correlation; r_p_, Phenotypic correlation; σ_g_, Genetic variance.

The ADG was weakly correlated to the rumen’s MESOR in WS (0.23) and FW (0.37). Negative correlations (*p* < 0.003) were observed between rectal amplitude with ruminal MESOR (−0.38) and with rectal MESOR (−0.40) in the FW, but the rectal MESOR showed a strong and negative correlation (−0.75) with rectal amplitude in the WS. There was no correlation between rumen MESOR with RBG during WS, but RFI showed a weak association with rumen MESOR in the FW.

The RBG and RIG showed similar correlations with FCE in both regimes (r > 0.83). As expected within both regimes, ADG showed strong positive correlations (r > 0.75) with FCE and RBG but no correlation with RFI. The ADG’s correlation with RIG was moderately high in both regimes (0.43–0.54) but was strong and negative with FCR in both regimes. While FCR was moderately and positively correlated with RFI, the correlation was stronger but negative with RBG and RIG. The MEI was strong and positive with RFI in both FW (0.71) and WS (0.78) but was moderate and negative with RIG in FW (−0.32) and WS (−0.46). The RFI was negatively correlated to RBG and RIG in both regimes, but RBG had a strong positive correlation with RIG.

The correlations between both regimes (the diagonal in Figure 3) were moderately strong (*p* < 0.001) for ruminal MESOR (0.58), stronger for rumen amplitude (0.85), but moderate for rectal MESOR (0.40), rectal amplitude (0.59), and rumen acrophase (0.63). There were moderate to low correlation between the two successive regimes for MEI, RBG, ADG, FCR, FCE and RIG.

### Genetic parameters for rhythm-adjusted core body temperature measures

There were higher heritability estimates (±PSD) in the FW than in the WS for the rumen (0.78 ± 0.18 vs. 0.50 ± 0.18) and rectal (0.56 ± 0.26 vs. 0.47 ± 0.22) temperatures. The heritability estimates for other circadian parameters in both regimes are shown in Table 4. The genetic correlation between the FW and WS measures were 0.69 ± 0.21 and 0.32 ± 0.59 for the rumen and rectal MESORs, respectively. Even though there was no phenotypic correlation between both regimes for the rectal MESOR (0.03 ± 0.17), the phenotypic correlation between both regimes for the rumen MESOR (0.63 ± 0.05) was moderately high, indicating appreciable predictability of performance from one regime to the other.

## Discussion

This applied research study evaluated the potential of predicting complex traits using novel phenotypes from cattle rumen boluses. The outcomes here can also support the collection of difficult-to-measure phenotypes or economic traits during winter, which is the longest season and potentially has the most inclement weather conditions in North America. The study evaluated the relationships between core body temperature and production efficiency measures by engaging two automatic data-logging devices to monitor beef steers’ rumen and rectal temperatures (every 5 min) over two successive winter-feeding regimes. We also evaluated the potential for circadian-adjusted parameters generated from these traits to become proxies for these production efficiency traits, especially in production settings.

The forage-based diets fed to the steers across the two phases in this study will supplement information on the repeatability of feed and growth efficiency traits measured across successive feeding periods (Durunna et al., 2011; Durunna et al., 2012). A commercial production efficiency test that provides the performance information (including individual feed intake and body gain) is cost-prohibitive, posing a significant barrier for producers interested in conducting multi-environment tests on their replacement candidates.

Understanding the relationships between CBT with production and efficiency traits in growing beef cattle managed under extensive conditions or in unshielded pens during winter is important. It may provide selection and management options that will help stockmen identify animals with better genetic potentials using less laborious and expensive tools. Applying such tool(s) in practical beef production environments will also create the opportunity for producers to pre-screen many replacement candidates (bulls or heifers) before conducting an actual feed test. Such pre-screening activities will help producers objectively assess a larger pool of potential candidates, thereby improving genetic progress through increased selection intensity.

This study observed that the average rumen and rectal temperatures were greater in the FW than in the WS, in converse with the average ambient temperature patterns. The results agree with Prendiville et al. (2002), who reported an inverse relationship between the ambient temperature with rumen (−0.63) and rectal (−0.42) temperature. Animals usually deploy autonomic and behavioural mechanisms that increase the internal temperature to compensate for the exposure of the skin to lower ambient temperature. Therefore, it is likely that deep thermoreceptors, which support maintaining thermoregulation (Werner et al., 2008; Taylor, 2014; Godyn et al., 2019), contributed to the inverse relationship between higher rumen temperature and season with lower ambient temperature. It is also likely that the need to balance the colder ambient temperature with a higher core temperature (e.g., through increased metabolic rate, activity, etc.) may manifest in the observed higher body temperature during the colder season or regime. Liang et al. (2013) reported that ambient temperatures increased the rumen temperatures in Holstein cows, where the summer rumen temperatures were higher than in other seasons.

Homeotherms (e.g., mammals and birds) can maintain a specific body temperature range, thereby regulating their daily rhythms (Aschoff 1982; Refinetti and Menaker 1992; Maloney et al., 2013). We do not know whether the same processes are responsible for the differences in the circadian temperature parameters. The changes in the daily CBT rhythm (e.g., in amplitude) could occur as a response to feed intake, given that some reports have shown that feed restriction or long-term lower feed intake is associated with lower morning rectal temperature in Sudanese goats (Ahmed and El Kheir 2004) or lower vaginal temperatures in Suffolk ewes (Sudarman and Ito 2000). Maloney et al., 2013 also showed that reduced energy intake could affect CBT during the inactive phase. Farm animals could have late acrophases (Refinetti, 2020) especially for those that are active into late hours. The acrophase or the peak time usually occur the animals are most active.

Further, the inability to periodically reassess these animals hinders our understanding of the repeatability of these traits across feeding regimes, seasons, or physiological/maturity stages (Durunna et al., 2011). The information about the relationships between data from automated or telemetric tools successively collected under typical feeding regimes and during the typical cold seasons and feeding environments will advance our knowledge of such tools. This information is important, especially in selection and breeding activities, which helps improve the competitiveness and profitability of the beef cattle sector.

Classifying animals into different efficiency classes creates a broad cohort of animals with similar characteristics, enabling livestock managers and breeders to know specific characteristics of that group. Even though several bovine studies have reported that rumen temperatures are excellent proxies for CBT (Hicks et al., 2001; Prendiville et al., 2002; Small et al., 2008), the lack of differences among temperature classes in this study indicates that neither the rumen nor rectal temperature parameters can be used as reliable indicators for predicting the production efficiency profile in growing cattle. However, the intricate nature of the trait, complexities of the breeds (crossbreds) in the industry, and the diversity of the feeding regimes and production systems may introduce some subtle differences, especially in small cohorts.

This study supported the evidence that RTMP is higher than RCT. Higher RTMP compared to RCT or ear temperature is due to the fermentation activities of rumen microbes (Hicks et al., 2001; Schutz and Bewley, 2009). The cooling effects of drinking bouts and rumen fermentation events may reduce the actual values (Kou et al., 2017). We also demonstrated that a strong correlation existed between RCT and RTMP. Bewley et al. (2008) showed that RTMP and RCT were strongly correlated (r = 0.64) in dairy cows, while Prendiville et al. (2002) reported a 0.34 correlation between the bolus and rectal temperature among paired rectal and ruminal data collected over 8 days across all seasons. A higher correlation (0.92) between RTMP and RCT was reported by Sievers et al., 2004 using 36 observations, while Edwards et al. (2002) demonstrated that gut temperatures were more closely related to rectal temperatures in human subjects’ axillary temperatures taken under the arm.

This study has established a moderately high (0.63) correlation between the two successive regimes for the rumen MESOR but none for rectal temperature. The lack of repeatability for the rectal temperature may be related to the interventions on individuals with protruding harnesses or direct exposure of the device to the elements that may affect the actual measurements in extreme cold. Unintended contacts of the exposed dataloggers with other objects or animals will affect the measurements. The location of the rectal device compared to the rumen boluses exposed them to more frequent intrusions from other animals or objects.

The RFI and RBG are measures of choice for assessing animals for feed or growth efficiency because they are independent of body weight and adjust for differences in the related energy sinks. While RFI considers the difference between the actual feed intake and predicted feed intake identifying animals that consume more or less feed than expected, the RBG profiles identify faster-growing animals without extra feed intake on the same body size (Detweiler et al., 2019). The more recent tool that draws from the desirable characteristics of both RFI and RBG is the residual intake and gain (RIG), which identifies animals with superior body gains at lower average feed intake (Berry and Crowley, 2012), thereby retaining the desirable characteristics of both traits. These production efficiency traits are expensive to measure, complex, and usually obtained once in the life of animals, which are usually replacement candidates. Our study showed that the temperature profiles cannot discriminate animals with different production efficiency profiles. The lack of differences among different efficiency classes within each feeding regime agrees with the observations of Lam et al. (2018), who reported no differences in the rumen temperature in feedlot cattle classified into an efficient or inefficient group based on standard deviation of RFI.

The correlation between the two regimes for RFI and RIG showed positive and moderate relationships between the two successive regimes. The repeatability estimates are in line with other studies for RFI (Kelly et al., 2010; Durunna et al., 2011; Durunna et al., 2012) across different diets. Potts et al. (2015) reported high RFI repeatability in dairy cows when fed high or low starch diets. Gomes et al. (2012) provided a mixed ration with about 11 MJ/kg energy density to steers over two successive periods, where period 2 in that study evaluated a subset of 12 efficient and 12 inefficient steers from a cohort of 72 steers. They reported a Spearman rank correlation of 0.40 and 0.11 for RFI and G:F, respectively. Russell et al. (2016) reported a low inter-period correlation for DM digestibility between two different successive feeding regimes using either a roughage-based diet or whole-shell corn-based in the growing and finishing phases that utilized cracked corn or products such as distiller’s grain and soybean hull. The repeatability across diets for DM digestibility ranged between 0.21 and 0.68 but G:F ranged between 0 and −0.57. The reasons behind the lack of repeatability for RBG, FCR, and FCE (in this study) may be related to the ADG component, which was also not repeatable.

The heritability estimates for the rumen and rectal MESORs were higher than the estimates reported by Dikmen et al. (2012) and Luo et al. (2021). The study reported low heritability estimates of 0.17 and 0.06, respectively, for the rectal temperatures measured in response to heat stress in Holstein cows. Sarlo-Davila et al., 2019 estimated heritability of 0.26 and 0.32 under high and low THI in crossbred beef heifers. Their reports agree with the higher heritability obtained in this study under the regime with lower ambient temperature. The higher estimates in this study may have benefited from a larger sample size, higher accuracy, and more consistent, uninterrupted measurement intervals. The higher heritability during the FW compared to the WS may be related to the innate ability of the steers to maintain a more consistent temperature during the FW. It could also be related to the colder environment under which the previous progenitors or generations were adapted and selected. Compared to the WS estimates, the high heritability estimates for the rumen and rectal temperature in the FW imply that direct selection based on the MESOR phenotypes will result in faster genetic progress of the associated traits if exploited in breeding programs.

## Data Availability

The raw data supporting the conclusion of this article will be made available by the authors, without undue reservation.
