# Corrigendum: Phenotypic and genetic parameters of circadian rhythms from core body temperature profiles and their relationships with beef steers’ production efficiency profiles during successive winter feeding periods

**DOI:** 10.3389/fgene.2023.1154825

**Published:** 2023-02-10

**Authors:** Obioha Durunna, Jeffery A. Carroll, Jeff W. Dailey, Daalkhaijav Damiran, Kathy A. Larson, Edouard Timsit, Rex Parsons, Ghader Manafiazar, Herbert A. Lardner

**Affiliations:** ^1^ Department of Applied Research, Lakeland College, Vermilion, AB, Canada; ^2^ Department of Animal and Poultry Science, University of Saskatchewan, Saskatoon, SK, Canada; ^3^ USDA ARS Livestock Issues Research Unit, Lubbock, TX, United States; ^4^ Department of Agricultural and Resource Economics, University of Saskatchewan, Saskatoon, SK, Canada; ^5^ Department of Production Animal Health, Faculty of Veterinary Medicine, University of Calgary, Calgary, AB, Canada; ^6^ Australian Centre for Health Services Innovation and Centre for Healthcare Transformation, School of Public Health and Social Work, Faculty of Health, Queensland University of Technology, Kelvin Grove, QLD, Australia; ^7^ Animal Science and Aquaculture Department, Faculty of Agriculture, Dalhousie University, Halifax, NS, Canada

**Keywords:** winter, core body temperature, reticulo-rumen, rectal, feed efficiency, telemetry, genetic parameters, multi-environment evaluations

In the published article, there was an error in the heading of column three of Table 1. The heading has been corrected to “Regime 2 moderate-forage diet.”

**TABLE 1:** Nutrient composition of diet fed to backgrounding steers during the Fall-Winter and Winter-Spring regimes over the 2 years of study.

Item	Regime 1 high-forage diet	Regime 2 moderate-forage diet
Dry matter (%)	80.67 ± 14.00	85.48 ± 12.00
Ash (%)	6.67 ± 0.29	8.60 ± 1.10
Crude fat (%)	1.27 ± 0.47	1.62 ± 0.29
Acid detergent fibre (%)	40.60 ± 0.86	37.89 ± 2.72
Neutral detergent fibre (%)	60.83 ± 2.08	56.54 ± 3.33
Non-structural carbohydrate profile (%)	16.32 ± 3.54	21.17 ± 3.69
Starch (%)	3.75 ± 0.94	7.08 ± 3.21
Crude protein (%)	11.35 ± 1.79	10.80 ± 1.83
Total digestible nutrients^a^, % DM	57.27 ± 0.67	59.38 ± 2.12
ME (mJ)^b^ = TDNx0.04409 × 0.82 × 4.184	8.66	8.98
Energy values (Mcal/kg DM)		
Net energy for gain	0.65 ± 0.02	0.72 ± 0.06
Net energy maintenance	1.37 ± 0.02	1.44 ± 0.06
Macro elements (%, DM)		
Calcium	0.64 ± 0.13	1.25 ± 0.35
Phosphorus	0.17 ± 0.04	0.19 ± 0.04
Potassium	1.56 ± 0.15	1.53 ± 0.32
Sulphur	0.15 ± 0.02	0.16 ± 0.01
Magnesium	0.21 ± 0.03	0.23 ± 0.04
Sodium	0.02 ± 0.01	0.14 ± 0.07
Micro minerals (g/kg DM)		
Zinc	15.27 ± 2.93	63.33 ± 27.33
Iron	85.32 ± 21.97	173.81 ± 106.09
Manganese	40.20 ± 5.84	68.20 ± 15.37
Copper	4.68 ± 0.35	15.71 ± 7.45

^a^ Calculated using the Weiss equation (1992).

^b^ ME, MJ∙kg^−1^ of DM = [(TDN, %/100) × 4.4 Mcal∙kg^−1^ of TDN] × 4.184 MJ of DE∙Mcal^−1^ × 0.82 MJ of ME∙MJ^−1^ of DE (National Research Council, 1996).

In the published article, there was an error in the caption of Table 3. The caption has been corrected to “production efficiency-based production efficiency-based classification (LSmeans) and circadian parameters for rumen and rectal temperature parameters calculated within the two regimes.”

**TABLE 3:** Production efficiency-based production efficiency-based classification (LSmeans) and circadian parameters for rumen and rectal temperature parameters calculated within the two regimes.

Variable	Efficient	Neutral	Inefficient	Class	Regime	Year	Class*Regime
RFI Classification							
Rumen Temperature							
Mean Temperature (MESOR)	39.72 ± 0.01	39.74 ± 0.01	39.75 ± 0.01	0.30	0.0004	<0.0001	0.72
Amplitude	0.26 ± 0.009	0.26 ± 0.008	0.27 ± 0.009	0.31	0.0003	0.005	0.03
Acrophase/Peak time	17.72 ± 0.19	17.32 ± 0.18	17.56 ± 0.19	0.03	<0.0001	0.01	0.31
Rectal Temperature							
Mean Temperature (MESOR)	39.11 ± 0.03	39.10 ± 0.03	39.15 ± 0.03	0.34	<0.0001	0.02	0.57
Amplitude	0.27 ± 0.01	0.29 ± 0.01	0.27 ± 0.01	0.56	0.08	0.31	0.09
Acrophase/Peak time	18.43 ± 0.25	18.15 ± 0.24	18.42 ± 0.24	0.51	0.0006	<0.0001	0.99
RBG Classification							
Rumen Temperature							
Mean Temperature (MESOR)	39.75 ± 0.01	39.74 ± 0.01	39.73 ± 0.01	0.64	0.0004	<0.0001	0.94
Amplitude	0.26 ± 0.009	0.27 ± 0.009	0.27 ± 0.009	0.03	<0.0001	0.004	0.44
Acrophase/Peak time	17.50 ± 0.18	17.52 ± 0.19	17.50 ± 0.19	0.98	<0.0001	0.01	0.94
Rectal Temperature							
Mean Temperature (MESOR)	39.12 ± 0.03	39.14 ± 0.03	39.11 ± 0.02	0.75	<0.0001	0.03	0.42
Amplitude	0.28 ± 0.01	0.26 ± 0.01	0.29 ± 0.01	0.26	0.07	0.51	0.18
Acrophase/Peak time	18.42 ± 0.24	18.22 ± 0.26	18.30 ± 0.34	0.78	0.0004	<0.0001	0.30
RIG Classification							
Rumen Temperature							
Mean Temperature (MESOR)	39.73 ± 0.014	39.74 ± 0.012	39.74 ± 0.014	0.88	0.0004	<0.0001	0.68
Amplitude	0.26 ± 0.009^ab^	0.26 ± 0.009^a^	0.27 ± 0.009^b^	0.03	<0.0001	0.003	0.17
Acrophase/Peak time	17.62 ± 0.19	17.40 ± 0.18	17.53 ± 0.20	0.32	<0.0001	0.01	0.22
Rectal Temperature							
Mean Temperature (MESOR)	39.10 ± 0.03	39.13 ± 0.03	39.13 ± 0.03	0.66	<0.0001	0.02	0.76
Amplitude	0.28 ± 0.01	0.26 ± 0.01	0.28 ± 0.01	0.23	0.03	0.41	0.12
Acrophase/Peak time	18.40 ± 0.24	18.42 ± 0.24	18.19 ± 0.23	0.61	0.0004	<0.0001	0.17

RFI, residual feed intake; RBG, residual body gain; RIG, residual intake and gain. FW, Fall-Winter; WS, Winter-Spring regime.

In the published article, there was an error in the caption of Table 4. The caption has been corrected to “Heritability (±PSD) and some genetic parameters for circadian core body temperature parameters.”

**TABLE 4:** Heritability (±PSD) and some genetic parameters for circadian core body temperature parameters.

Variable	FW h^2^	HPD95	WS h^2^	HPD95	FW σg	WS σg	r_g_	r_p_
Rumen MESOR	0.78 ± 0.18	0.44–0.99	0.50 ± 0.18	0.17–0.85	0.013	0.007	0.69 ± 0.21	0.63 ± 0.05
Rectal MESOR	0.56 ± 0.26	0.09–0.99	0.47 ± 0.22	0.07–0.87	0.01	0.02	0.32 ± 0.59	0.03 ± 0.17
Bolus Amplitude	0.56 ± 0.25	0.13–0.99	0.39 ± 0.19	0.05–0.76	0.0008	0.0007	0.23 ± 0.52	0.41 ± 0.07
Rectal Amplitude	0.52 ± 0.27	0.06–0.98	0.42 ± 0.21	0.04–0.80	0.001	0.01	0.40 ± 0.59	-0.01 ± 0.16
Bolus Acrophase	0.17 ± 0.17	0.00002–0.54	0.34 ± 0.18	0.02–0.69	0.31	0.28	0.53 ± 0.59	0.30 ± 0.08
Rectal Acrophase	0.32 ± 0.25	0.0001–0.83	0.50 ± 0.22	0.12–0.91	0.62	1.26	0.14 ± 0.66	0.40 ± 0.13

FW, Fall-Winter regime; WS, winter spring; h^2^, heritability; HPD95, Highest posterior density intervals; r_g_, Genetic correlation; r_p_, Phenotypic correlation; σ_g_, Genetic variance.

The corrected Tables 1, 3, 4 appear below.

A correction has been made to **Results**, Paragraph 3. This sentence previously stated:

“There was no regime-by-year interaction (*p* > 0.05) for the average age and weight at the initiation of the study, DMI, ADG, and for all temperature parameters except the acrophase of rumen MESOR (*p* < 0.0001). As expected, the DMI, MEI, and ADG were greater (*p* < 0.0001) in the WS compared to the FW.”

The corrected sentence appears below:

“There was no regime-by-year interaction (*p* > 0.05) for the average age and weight at the initiation of the study, DMI and for all temperature parameters except the acrophase of rumen MESOR (*p* < 0.0001). As expected, the DMI and MEI were greater (*p* < 0.0001) in the WS compared to the FW.”

A correction has been made to **Results**, Paragraph 4. This sentence previously stated:

“The rumen MESORs were 39.75°C, 39.65°C, 39.82°C, and 39.75°C, respectively, for Yr1 regime1, Yr1 regime2, Yr2 regime1, and Yr2 regime2.”

The corrected sentence appears below:

“The rumen MESORs were 39.75°C, 39.65°C, 39.82°C, and 39.75°C, respectively, for Yr1 FW, Yr1 MS, Yr2 FW, and Yr2 MS.”

A correction has been made to **Results**, *Relationships among the traits and parameters within and between the two regimes*, Paragraph 1. This sentence previously stated:

“Table 4 shows the correlations among production traits and rhythm-adjusted temperature parameters within each test-regime, and between the two regimes.”

The corrected sentence appears below:

“**Figure 3** shows the correlations among production traits and rhythm-adjusted temperature parameters within each test-regime, and between the two regimes.”

A correction has been made to **Results**, *Relationships among the traits and parameters within and between the two regimes*, Paragraph 1. This sentence previously stated:

“Within the FW (above the diagonal), a strong positive correlation (*p* < 0.0001) was observed between the MESORs for rumen and rectal temperature (0.74) but was weaker (0.30; *p* = 0.02) and moderate between rumen amplitude and rectal amplitude (0.42). The MEI was moderately positively correlated with rumen MESOR (0.38) but weaker with rectal MESOR (0.21) during the FW.”

The corrected sentence appears below:

“Within the FW (above the diagonal), a strong positive correlation (*p* < 0.0001) was observed between the MESORs for rumen and rectal temperature (0.74) but was moderate between rumen amplitude and rectal amplitude (0.42; *p* < 0.001). The MEI was moderately positively correlated with rumen MESOR (0.38; *p* < 0.0001) but weaker with rectal MESOR (0.21; *p* < 0.10) during the FW.”

A correction has been made to **Results**, *Relationships among the traits and parameters within and between the two regimes*, Paragraph 2. This sentence previously stated:

“The correlations between both regimes (the diagonal in **Figure 3**) were moderately strong (*p* < 0.0001) for ruminal MESOR (0.58), stronger for rumen amplitude (0.85), but moderate (*p* > 0.60) for rectal MESOR (0.40), rectal amplitude (0.59), and rumen acrophase (0.63).”

The corrected sentence appears below:

“The correlations between both regimes (the diagonal in **Figure 3**) were moderately strong (*p* < 0.001) for ruminal MESOR (0.58), stronger for rumen amplitude (0.85), but moderate for rectal MESOR (0.40), rectal amplitude (0.59), and rumen acrophase (0.63).”

A correction has been made to **Results**, *Genetic parameters for rhythm-adjusted core body temperature measures*, Paragraph 1. This sentence previously stated:

“Even though there was no phenotypic correlation between both regimes for the rectal MESOR (0.03 ± 0.17), the phenotypic correlation between both regimes for the rumen temperature (0.63 ± 0.05) was moderately high, indicating appreciable predictability of performance from one regime to the other.”

The corrected sentence appears below:

“Even though there was no phenotypic correlation between both regimes for the rectal MESOR (0.03 ± 0.17), the phenotypic correlation between both regimes for the rumen MESOR (0.63 ± 0.05) was moderately high, indicating appreciable predictability of performance from one regime to the other.”

A correction has been made to **Discussion**, Paragraph 6. This sentence previously stated:

“The information about the relationships between data from automated or telemetric tools successively under typical feeding regimes and during the typical cold seasons and feeding environments will advance our knowledge of such tools.”

The corrected sentence appears below:

“The information about the relationships between data from automated or telemetric tools successively collected under typical feeding regimes and during the typical cold seasons and feeding environments will advance our knowledge of such tools.”

A correction has been made to **Discussion**, Paragraph 8. This sentence previously stated:

“We also demonstrated a strong correlation between RCT and RTMP.”

The corrected sentence appears below:

“We also demonstrated that a strong correlation existed between RCT and RTMP.”

A correction has been made to **Discussion**, Paragraph 12. This sentence previously stated:

“The high heritability estimates for the rumen and rectal temperature in the FW imply that direct selection based on the MESOR phenotypes will result in faster genetic progress of associated traits if exploited in breeding programs.”

The corrected sentence appears below:

“Compared to the WS estimates, the high heritability estimates for the rumen and rectal temperature in the FW imply that direct selection based on the MESOR phenotypes will result in faster genetic progress of the associated traits if exploited in breeding programs.”

The authors apologize for this error and state that this does not change the scientific conclusion of the article in any way. The original article has been updated.

